# Airway-centered interstitial fibrosis: etiology, clinical findings and prognosis

**DOI:** 10.1186/s12931-015-0213-7

**Published:** 2015-05-09

**Authors:** Lilian Tiemi Kuranishi, Kevin O Leslie, Rimarcs Gomes Ferreira, Ester Aparecida Ney Coletta, Karin Mueller Storrer, Maria Raquel Soares, Carlos Alberto de Castro Pereira

**Affiliations:** Pulmonary Department, Federal University of São Paulo, Sao Paulo, Brazil; Pathology Department, Mayo Clinic, Scottsdale, USA; Pathology Department, Federal University of São Paulo, Sao Paulo, Brazil

**Keywords:** Interstitial lung disease, Hypersensitivity pneumonia, Gastroesophageal reflux, Pulmonary fibrosis

## Abstract

**Background:**

Airway-centered Interstitial Fibrosis (ACIF) is a common pathologic pattern observed in our practice.

**Objectives:**

The objectives of this study are to describe the causes associated with ACIF in a large sample of patients and its effect on survival.

**Methods:**

A retrospective study in three centers of interstitial lung disease in São Paulo, between January of 1995 and December of 2012. The surgical lung biopsy specimens were reviewed by three pathologists. The clinical, functional and tomographic findings were analyzed by a standardized protocol.

**Results:**

There were 68 cases of ACIF, most of them women. The mean age was 57 ± 12 yr. Dyspnea, cough, restrictive pattern at spirometry and oxygen desaturation at exercise were common. A reticular pattern with peribronchovascular infiltrates was found in 79% of the cases. The etiologies of ACIF were hypersensitivity pneumonitis in 29 (42.6%), gastroesophageal reflux disease in 17 (25.0%), collagen vascular disease in 4 (5.9%), a combination of them in 15 cases and idiopathic in 3 (4.4%). The median survival was 116 months (95% CI = 58.5 – 173.5). Lower values of oxygen saturation at rest, presence of cough and some histological findings - organizing tissue in the airways, fibroblastic foci and microscopic honeycombing - were predictors of worse survival.

**Conclusions:**

ACIF is an interstitial lung disease with a better survival when compared with IPF. The main etiologies are HP and GERD. The oxygen saturation at rest, the presence of cough and some histological findings are predictors of survival.

## Introduction

Since 2002 a new interstitial pneumonia centered on small airways and no granulomas has been described in small series [1-4]. This entity has been variably called centrilobular fibrosis, bronchiolocentric interstitial pneumonia, airway-centered interstitial fibrosis, and peribronchiolar metaplasia [3-6]. Lung biopsies from these cases showed a distinctive pattern of interstitial fibrosis centered and extending around the bronchioles, often with bronchiolar metaplasia of the epithelium. Due to involvement of small as well large airways [5,7], the best name seems to be airway-centered interstitial fibrosis (ACIF). The prognosis is unknown due to small number of cases described [2-4]. The etiology in these series was unclear. Similar pathologic findings have been described in hypersensitivity pneumonitis (HP) [8-15] or be secondary to gastroesophageal reflux disease (GERD), isolated or associated with connective tissue diseases [16,17]. In our clinical practice this pathologic pattern is common in multidisciplinary case discussions.

The objectives of the present study were to describe the causes, the clinical, tomographic, functional and pathologic findings and their influence on survival in a large number of patients with histologic diagnosis of airway-centered interstitial fibrosis.

## Material and methods

### Selection of cases

A retrospective cohort of 68 adult patients with ACIF was evaluated. The medical records of 2716 patients with interstitial lung diseases, seen between January of 1995 and December of 2012 at three facilities in the city of São Paulo, Brazil, were reviewed. From this register 600 patients were submitted to surgical lung biopsies. Cases with any findings of possible ACIF or describing bronchiolar involvement without a specific diagnosis of bronchiolitis were reevaluated. In this database, 115 patients had a diagnosis of possible ACIF by surgical lung biopsy. From this sample, 36 cases were excluded due to a another diagnosis after a review of the slides (mainly bronchiolitis, characterized by bronchiolar inflammation and/or fibrosis in the absence of peribronchiolar involvement as well absence of reticular pattern on HRCT); six cases were excluded due to incomplete data and five due to honeycombing (an exclusion criteria due to possible non representative biopsies in advanced disease) observed on a HRCT scan. None of the patients died after lung biopsy. Biopsies were done by small thoracotomies or VATS, involving one or more sites, but this information was not available in final analysis. The final sample comprised 68 patients.

This study was approved by ethics committee of the Federal University of São Paulo (register number 2079-09).

### Histological findings

Patients were selected primarily by a revision of lung biopsies by two pathologists dedicated to lung pathology, and with a large experience in interstitial lung disease (ILD). The cases were selected initially by one of them and 48 of them were sent to KOL for confirmation. He agreed with diagnoses and described and classified the findings in each case. The remaining twenty cases were diagnosed by consensus between our two pathologists, who also followed the same classification of findings. Concordance by kappa was not calculated. The diagnosis was based on previously suggested criteria [4-6]. The main diagnostic criteria included a fibrosis predominantly bronchiolocentric associated with bronchiolar or peribronchiolar inflammation and peribronchiolar metaplasia. Cases with granulomas and foreign material were excluded. Incidental findings like such as fibromyxoid tissue foci in airways, fibroblastic foci, honeycombing, giant cells, cholesterol clefts, respiratory bronchiolitis, features of acute injury (tissue edema and the presence of fibrin), and focal areas of heterogeneous or homogeneous fibrosis (usual interstitial pneumonia (UIP) and nod-specific interstitial pneumonia (NSIP) like, respectively) were recorded.

### Clinical analysis and HRCT

A standardized protocol for investigation of interstitial lung diseases was used for all patients. Dyspnoea was assessed by Magnitude of Task of Basal Dyspnea Index (BDI) [18]. Total BDI score was not considered because functional impairment and magnitude of effort do not involve the same activities in different patients. The data related to environmental exposures were recorded. A diagnosis of HP was based on the exposure and the absence of other potential causes of ACIF. Precipitins tests and cellular analysis of bronchoalveolar lavage were not available. Possible connective tissue disease (CTD) was investigated by clinical and complementary tests in all cases. The diagnostic criteria for CTD were those previously described [19].

Symptoms of GERD (heartburn, regurgitation) were recorded in all cases. An ambulatory 24-hour esophageal pH measurement with a dual sensor was performed according to standardized techniques [20]. The diagnostic criteria for an abnormal proximal [21] and distal [22] reflux have been previously described.

After biopsies selection, the final clinical diagnoses were retrospectively determined by multidisciplinary discussion. The diagnoses were classified as HP, GERD, CTD, a combination of two or more categories or idiopathic.

Pulmonary function tests were conducted according to the American Thoracic Society guidelines [23]. The normal values for spirometry were recalculated according 2007 values derived for the Brazilian population [24]. Normal values for DLco were from Crapo [25]. The peripheral oxygen saturation was evaluated at rest and after a 4-minute self-paced step test [26].

All CT scans were read by experienced radiologists and pulmonologists through a systematic analysis of the findings. The final findings were selected by consensus. Scores for extension of disease were not calculated. Expiratory views were not done systematically.

The drugs used for at least three months for ACIF and GERD treatment were recorded. Antigen avoidance and abatement procedures were recommended when exposure was present.

### Survival

Survival was assessed from the day of the biopsy through December 2012. Deaths were identified by a follow-up contact or through telephone notification by relatives. Deaths were considered ACIF-related if they were due to respiratory failure, pneumonia or pulmonary fibrosis. One patient was censored at the time of lung transplantation.

### Statistical analysis

All of the data analyses were performed using the SPSS program, version 19. According distribution, data were expressed as mean ± SD, or as median and range. The continuous data with a normal distribution were compared using t-tests. A chi-square test was used for comparisons of proportions. The impact of clinical, functional, tomographic and pathologic data on survival was calculated by univariate Cox regression ant by Kaplan-Meyer curves. Two-sided p values <0.05 were considered to be statistically significant. The study design was approved by the ethics committees of the hospitals involved.

## Results

The study sample comprised 68 cases, with a predominance of non-smokers, and females. The baseline characteristics are shown in Table 1. The main clinical features were cough (in 78%) and dyspnea (100%). The functional profile was typical of diffuse restrictive lung disease, with reduced FVC, FEV_1_, DL_CO_ and a decrease of SaO_2_ upon exercise. A reticular pattern, suggesting fibrosis, was present in all of the cases. Central and peribronchovascular distributions were seen in 79% of the cases, but an associated peripheral distribution was common.

**Table 1:** **Baseline characteristics of patients with airway-centered interstitial fibrosis (n = 68)**

**Characteristic**	**Number**
Sex, male/female	29/39
Age, years (mean ± SD)	57 ± 12
Duration of symptoms, in months, median (range)	24 (3–132)
Smoking	
Never/ex-smoker/current smoker, n	39/28/1
Dyspnea	
Major/moderate/light tasks, n	22/33/13
Coughing, n (%)	53 (78%)
Clubbing, n (%)	12 (18%)
Velcro crackles, n (%)	29 (43%)
Exposure to organic particles, n (%)	42 (62%)
Molds/birds/both	24/ 4/ 14
Gastroesophageal reflux (GERD) symptoms	
None/past/current	30/ 12/ 26
Connective tissue disease (CTD), n (%)^†^	12 (17%)
Lung function tests	
FVC, % predicted (mean ± SD)	66 ± 18
FEV_1_, % predicted (mean ± SD)	69 ± 18
FEV_1_/FVC, (mean ± SD)	0.85 ± 0,08
DL_CO_, % predicted (mean ± SD) (n = 43)	59 ± 17
Oxygen saturation at rest, % (mean ± SD)	94 ± 4
Oxygen saturation during exercise, % (mean ± SD) (n = 59)	87 ± 7
HRCT findings	
Reticular infiltrate, n (%)	68 (100%)
Predominance: Upper lobe/lower lobe/diffuse, n	10/38/20
Predominance: Central/peripheral/both, n	9/14/45
Ground-glass opacities, n (%)	57 (84%)
Peribronchovascular, n (%)	54 (79%)
Bronchiectasis, n (%)	43 (63%)
Mosaic pattern/airtrapping, n (%)	25 (37%)
Centrilobular nodules, n (%)	14 (21%)

^†^Systemic sclerosis (2), Rheumatoid arthritis (3), Mixed connective tissue disease (3), Dermatomyositis (2), Antisynthetase syndrome (1), Systemic sclerosis and Sjögren’s syndrome (1), FEV_1_ = forced expiratory volume in 1 s; FVC = forced vital capacity; DL_CO_ = monoxide carbon lung diffusion. HRCT = high resolution chest tomography.

Exposure to birds, molds or both was present in 42 (62%) of the cases. Current or past gastroesophageal reflux symptoms were reported by 56% of the cases. Ambulatory esophageal pH monitoring was carried on in 38 cases, and showed abnormal reflux (distal, proximal or both), in 28 (74%). In these 28 cases, past or present GERD symptoms were absent in 6 (21.4%). There was an association between order for ambulatory pH monitoring and reflux symptoms. Of the 30 patients not submitted to ambulatory pH monitoring, only 5 (16.7%) had current symptoms of GERD, compared with 21 (55.3%) of 38 patients submitted to pH monitoring (x^2^ = 10.58, p = 0.005). Esophageal manometry was performed in 32 patients and showed hypotonia of the upper esophageal sphincter, esophageal body or lower esophageal sphincter in 16 (50%).

**Figure 1:**
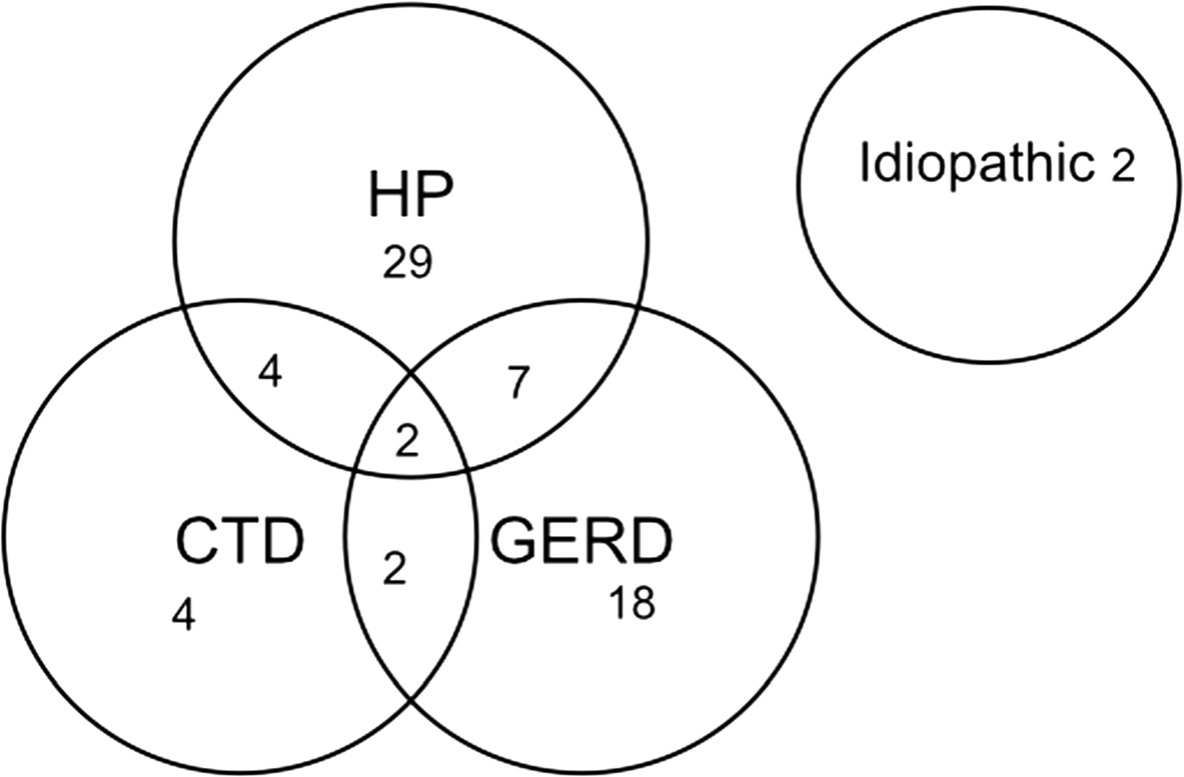
Clinical diagnoses of patients with airway-centered interstitial fibrosis (n = 68).

The final clinical diagnoses are shown in Figure 1. Hypersensitivity pneumonitis, GERD and CTD were the most common diagnoses. In only two cases an etiology was not apparent. The frequency of HRCT findings, including air trapping and centrilobular nodules did not differ between HP and GERD.

The histological findings observed are shown in Table 2. Peribronchiolar metaplasia was present in nearly 90% of the cases. Fibroblastic foci were observed in half of the cases. Microscopic honeycombing was found in 29% of the cases.

**Table 2:** **Histological findings in surgical lung biopsies from patients with airway-centered interstitial fibrosis (n = 68)**

**Major findings**	**n (%)**
Airway-centered interstitial fibrosis, n (%)	68 (100%)
Airway inflammation, n (%)	67 (98.5%)
Peribronchiolar metaplasia, n (%)	60 (88.2%)
Other findings (focal)	n (%)
Organizing tissue in airways, n (%)	25 (36.8%)
Giant cells, n (%)	12 (17.6%)
Interstitial heterogeneous fibrosis, n (%)	18 (26.5%)
Interstitial homogeneous fibrosis, n (%)	32 (47.1%)
Fibroblastic foci, n (%)	34 (50.0%)
Microscopic honeycombing, n (%)	20 (29.4%)

**Figure 2:**
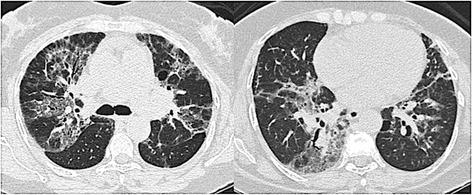
Radiologic findings of a patient with ACIF, showing diffuse ground glass opacities on peribronchovascular region, reticular infiltrates and traction bronchiectasias.

**Figure 3:**
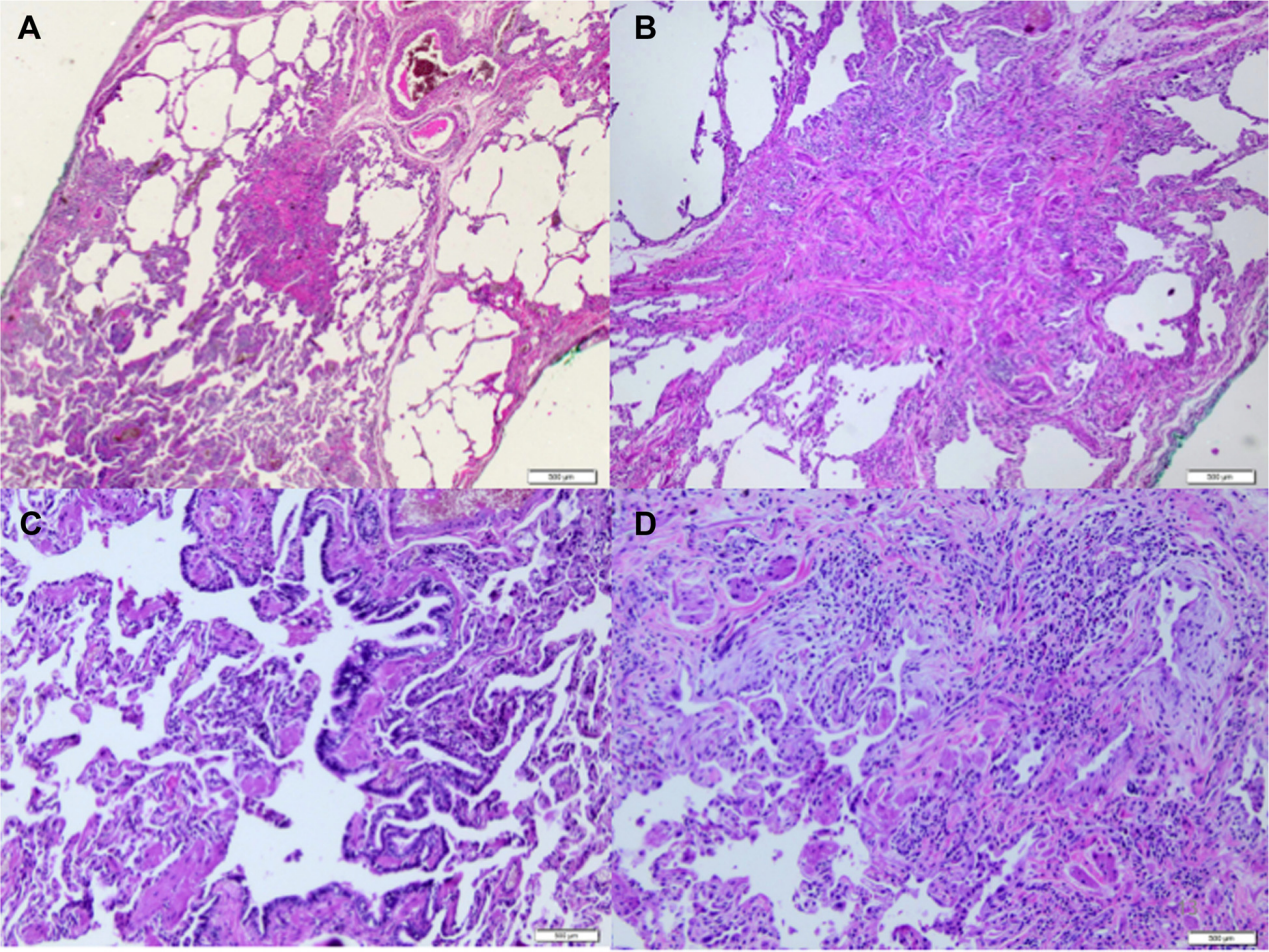
Histologic features of airway-centered interstitial fibrosis. **(A)** Airway-centered fibrosis with obliteration of bronchioles, **(B)** a detailed centrilobular fibrosis from figure a. **(C)** Peribronchiolar alveolar metaplasia. **(D)** Inflammatory peribronchiolar infiltration, obliterative focal organization on airways.

Images and histologic features in a representative case of ACIF are shown in Figures 2 and 3 respectively.

**Figure 4:**
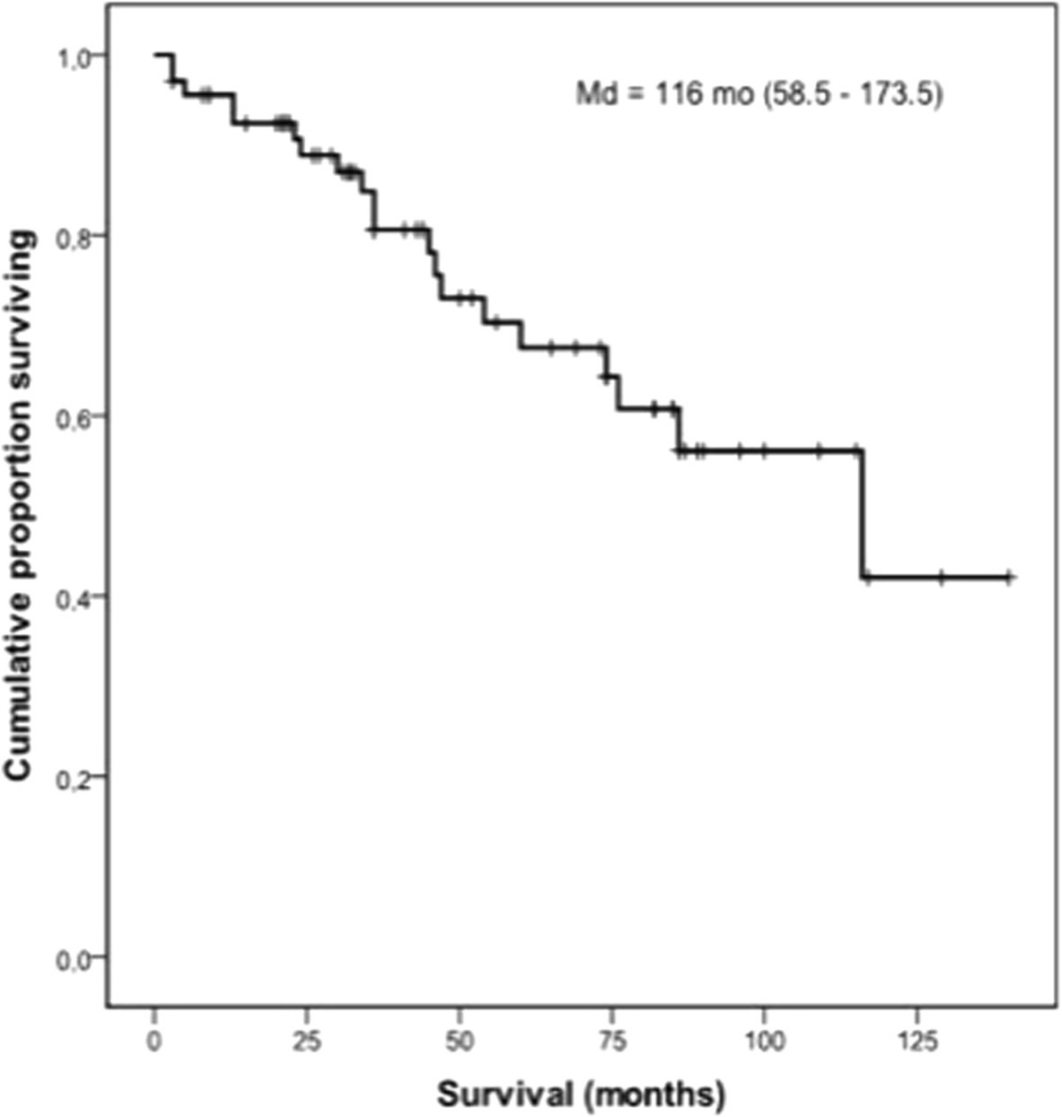
Kaplan-Meier survival curve for patients with airway-centered interstitial fibrosis (n = 68); median survival = 116 months (95% CI = 58.5 - 173.5).

The median post-biopsy follow-up period was 43.5 months. Kaplan-Meier survival curve for all cases is shown in Figure 4. The median survival was 116 months (95% CI = 58.5 – 173.5). Mortality after 5 years was 32.5%. By Cox analysis, survival was not influenced by sex, age, dyspnea, clubbing, crackles, FVC, DL_CO_, exercise SaO_2_, etiology and HRCT findings. Significant predictors of greater mortality by univariate analysis (p ≤ 0.10) were as follows: cough, lower SaO_2_ at rest and the presence of organizing tissue in airways, fibroblastic foci and microscopic honeycombing in lung biopsies (Table 3).

**Table 3:** **Univariate Cox analysis for significant survival predictor variables (p < 0.10) in patients with airway-centered interstitial fibrosis (n = 68)**

**Variable**	**HR**	**95% CI**	**p**
Cough	6.45	0.85-47.6	0.071
Oxygen saturation at rest	1.14	1.04-1.24	0.003
Organizing airway tissue	2.71	1.10-6.67	0.029
Fibroblastic foci	3.32	1.19-9.26	0.021
Microscopic honeycombing	2.76	1.07-7.14	0.036

The patients with cough (n = 53) had a median survival of 86 months; in those without cough (n = 15), the median survival was undetermined (log-rank = 4.30, p = 0.039). At the end of the follow-up, 1 of 15 (7%) patients without cough had died compared to 19 out of 53 (36%) patients with cough. The presence of cough was not associated with reflux symptoms or abnormal reflux by pH monitoring (data not shown). The patients with cough had a lower SaO_2_ at rest (93.7 ± 4.4 vs 96.0 ± 2.4, t = 1.93, p = 0.058) and at the end of exercise (85.6 ± 6.8 vs 91.3 ± 4.6, t = 3.00, p = 0.004). The FVC was lower in the patients with cough, but the difference was not significant (64.6 ± 18.2 vs 69.9 ± 20.2, p = 0.338). The patients with cough more commonly had fibroblastic foci in lung biopsies (58%), when compared with those without cough (20%) (x^2^ = 6.93, p = 0.008).

The rest SaO_2_ had an influence on survival (HR for decreasing values 1.14, 95% CI = 1.05-1.24, p = 0.003). For lung biopsies, the patients with microscopic honeycombing had an unfavorable prognosis. The median survival time was 45.0 (95% CI = 30.5-59.5) months in the 20 patients with microscopic honeycombing, in comparison to 116.0 (95% CI = 64.6-167.4) months for the 48 patients without honeycombing (log rank = 4.80, p = 0.028).

In the lung biopsies, a significant difference in survival was also observed when the patients with fibroblastic foci were compared with those without such findings. The median survival time was 74.0 (95% CI = 41.2-106.7) months in 34 patients with fibroblastic foci, compared with 116.0 months (95% CI = 58.5-173.5) in the 34 patients without fibroblastic foci (log rank = 5.98, p = 0.014).

The patients who had organizing tissue present in the airways had also a worse prognosis. The median survival time was 74.0 (95% CI = 23.8-124.1) months in 25 patients with organizing tissue in the airways, compared with 116.0 months (95% CI = 59.6-172.4) in the 43 patients without organizing tissue in airways (log rank = 4.80, p = 0.028).

Isolated giant cells were found in 12 cases - 4 with HP, 5 with aspiration, 2 with CTD and 1 with a combination of etiologies. Antigen avoidance and abatement procedures were recommended for all cases exposed to organic particles. GERD treatment was prescribed for all patients with abnormal in pH monitoring results or with current reflux symptoms. Pharmacological treatment was prescribed in 53 cases - corticosteroids in 23, isolated immunossupressor in 1, and both corticosteroids and immunossupressors in 29. The effect of these treatments on survival was uncertain.

## Discussion

The present study describes 68 patients with bronchiolocentric interstitial fibrosis. Hypersensitivity pneumonitis and GERD were the most common etiologies. The median survival was approximately 10 years but could be better predicted by histological findings.

In 2002 Yousem and Dacic [4] reported 10 patients with fibrosis and inflammatory process centered in small airways. In 2004, Churg et al. [5] described 12 patients with a more severe small bronchiolocentric interstitial fibrosis. Metaplastic bronchiolar epithelium extending around the bronchioles was described in both studies [4,5]. Because there was radiographic evidence of fibrosis around the large airways and microscopic evidence of fibrosis around the small airways, Churg et al. called this condition “Airway-centered Interstitial Fibrosis. Fukuoka et al. [6] described a histological pattern characterized essentially by the presence of peribronchiolar metaplasia in 15 patients.

Based on these studies, we selected the presence of bronchiolar-centered interstitial fibrosis, bronchiolar/peribronchiolar inflammation and peribronchiolar metaplasia as the major criteria for ACIF diagnosis. Peribronchiolar metaplasia is an incidental finding observed in a variety of ILDs, but in ACIF, the lesions are very conspicuous and findings indicative of other interstitial lung diseases are absent. There was no association between peribronchiolar metaplasia and smoking (data not shown).

The lesions centered in the airways suggest an injury due to inhalation or aspiration. In HRCT, peribronchial interstitial thickening and signs of bronchiolar involvement are consistent with this hypothesis. In our series, exposure to birds, molds or both at home, were common. Chronic interstitial pneumonia, bronchiolitis and a distinctive form of peribronchiolar granulomatous inflammation is the most frequent combination of findings in surgical lung biopsies from patients with a clinical diagnosis of HP [27], but granulomas can be absent, especially in chronic disease [8,9]. ACIF has been described in a subset of patients in small series and case reports of HP [7-10] and in case reports in which birds and molds have been well documented as the etiology [7,12-14]. Studies made in Japan have clearly shown that bronchiolocentric interstitial fibrosis is a common pathologic expression of chronic hypersensitivity pneumonitis [28-30]. Fibroblastic foci, honeycombing and organizing pneumonia were common. Granulomas were absent in many cases [28,29]. Bridging fibrosis between respiratory bronchioles and adjacent subpleural or intralobular septa seems to be a distinctive feature [28,29], but in our study this was not evaluated.

The aspiration of large volumes of gastric content to the lungs can result in several patterns of injury [31]. Inflammation, scarring and bronchiolar metaplasia in the centrilobular zones of the lung were identified in cases of chronic aspiration in humans [16] and in an experimental model [32]. Microaspiration of the gastric contents has been extensively evaluated in patients with IPF and in systemic sclerosis and other CTDs [31]. In scleroderma, a disease in which GERD is common, centrilobular fibrosis was described in 21% of 28 lung biopsies [17].

Chronic HP can result several histological patterns, including NSIP and an UIP-like pattern [8,9,15,28,33]. The findings of giant cells or granulomas in these cases could suggest the presence of HP. The presence of giant cells was observed in 18% of our cases, but was not specific to HP. Patients with granulomas were excluded from our study. Fibroblastic foci and honeycombing are both common in UIP, but can also be found in chronic HP [15,28,29,10,11]. IPF is a disease of older males, whereas ACIF predominates in women with a lower mean age. The prognosis of IPF is dire, with a median survival of 4 years after the diagnosis [34]. In our study, the median survival was 10 years, but when honeycomb changes were present in biopsies, the median survival was 45 months. The presence of fibroblastic foci was also predictive of worse survival, a finding similar to IPF [35].

Some conditions can result in bronchiolocentric lesions associated with fibrosis. A smoking-related fibrosis has been described under different names and a bronchiolocentric fibrosis can be observed [36,37]. In our study, as expected, focal respiratory bronchiolitis was found in some smokers, but other findings of smoking-related interstitial fibrosis were absent. ACIF could represent a late stage of a preexisting obliterans organizing pneumonia. Organizing tissue in airways was seen in 37% of our cases, but focal organizing pneumonia was seen in only one case, diagnosed as chronic hypersensitivity pneumonitis.

From a morphologic perspective, bronchiolocentric interstitial pneumonia needs to be separated from bronchiolitis [4]. In fact, several cases diagnosed as possible ACIF were excluded after review of slides and HRCT. Absence of peribronchiolar parenchymal lesions in biopsies and presence of diffuse reticular opacities on HRCT were major findings in differential diagnosis. Katzenstein *et al.* reported cases of nonspecific interstitial pneumonia with a bronchiolocentric distribution; however, several cases had relevant exposure and giant cells in biopsies [38]. Bronchiolocentricity of inflammation or fibrosis was seen in 9 (13.4%) cases in Travis et al. series of non-specific interstitial pneumonia, but when present it was not a dominant feature [39]. In the present study, bronchiolocentric lesions were dominant, and when present, areas of uniform fibrosis were focal.

In the recent ATS/ERS update on IIP classification, ACIF was not included because of questions concerning whether they are variants of existing IIPs or exist only in association with other conditions such as HP or CTD [40]. In our series, only two were considered to be idiopathic.

There are several limitations in our study. It was retrospective; although gold standards tests for the diagnosis of aspiration are not available, esophageal pH measurements were not performed in all cases. The diagnosis of HP was based on environmental exposures only. BAL cell count and precipitins were not available. A recent study from Japan on chronic HP found centrilobular fibrosis as the major pathologic finding in chronic HP. Elevated lymphocytes in BAL were absent in many cases [30].

In our study, agreement between readers of pathologic slides and HRCT scans was not examined. Five patients with honeycombing at HRCT were excluded, but 29% of cases had microscopic honeycombing. The cases excluded had etiology similar to those included. Extent of lung fibrosis on the baseline CT is predictive of survival in chronic HP [41], but extent of fibrosis on HRCT in our study was not evaluated. Finally, the influence of antigen avoidance, reflux treatment, and use de anti-inflammatory drugs on survival is unknown.

In conclusion, ACIF is common in HP, but can be found in other settings, especially microaspiration. Idiopathic disease is rare. The prognosis is more favorable than that published for IPF, but is reduced in patients with cough and in those patients with biopsies showing fibroblastic foci or organizing tissue in the airways, and especially when microscopic honeycombing is present.

## Abbreviation

ACIF: Airway-centered interstitial fibrosis
IPF: Idiopathic pulmonary fibrosis
HP: Hypersensitivity pneumonia
GERD: Gastroesophageal reflux disease
HRCT: High resolution computerized tomography
VATS: Video-assited thoracic surgery
ILD: Interstitial lung disease
UIP: Usual interstitial pneumonia
NSIP: Non-specific interstitial pneumonia
BDI: Basal dyspnea index
CTD: Connective tissue disease
BAL: Bronchoalveolar lavage
